# Pickup and delivery planning for the crowdsourced freight delivery routing problem

**DOI:** 10.1371/journal.pone.0318432

**Published:** 2025-02-24

**Authors:** Jingxian Zhang

**Affiliations:** 1 School of Management, Huazhong University of Science and Technology, Wuhan, Hubei Province, China; Wuhan Textile University, CHINA

## Abstract

Pickup and delivery problem (PDP) and dynamic vehicle routing problem (DVRP) are two key components of crowdsourced freight delivery services. Although previous research has focused predominantly on static vehicle routing problems, this study formally defines the dynamic problem specific to crowdsourced freight delivery and presents a mixed-integer linear programming model based on a rolling-horizon framework. The objective is to minimize total service costs, including fixed vehicle costs, transportation costs, and penalty costs for delays, while planning routes that cover all orders. To solve this combinatorial optimization problem, we propose an improved partheno genetic algorithm (IPGA) and a simulated annealing algorithm (SA). Numerical experiments demonstrate that the IPGA outperforms the SA, reducing the total service costs by over 10% on average. In addition, a real-world case study illustrates the practical applicability of our model and algorithms, providing a solid foundation for real-world implementation.

## 1 Introduction

With the rapid rise of e-commerce and the profound impact of the COVID-19 pandemic on global consumer shopping habits, competition in the logistics industry has become increasingly fierce, especially in terms of improving last-miles delivery efficiency [1,2]. To enhance competitiveness, renowned online retailers and logistics companies are actively exploring innovative delivery systems to achieve faster and more cost-effective delivery. One of such systems is “crowdsourced freight delivery”, where outsourced drivers carry out last-mile deliveries with their own vehicles, from designated pickup points to customers’ destinations. This service not only improves the last-miles delivery efficiency for users, but also creates job opportunities for millions of local drivers [3]. In recent years, the demand for crowdsourced freight delivery services has surged in China, exemplified by companies such as Dada Delivery [4], which specializes in on-demand city deliveries, and Huolala Logistics [5], which focuses on urban logistics. A similar trend is occurring in North America, where Amazon and Walmart operate their own crowdsourced delivery platforms known as Amazon Flex and Walmart Spark Delivery [6,7]. In addition, the number of companies participating in crowd-sourced freight delivery is rapidly growing, including BuddyTruk, Deliv, DHL MyWays, DoorDash, Hitch, Kanga, L Truxx, Nimber, PiggyBee, Postmates, Roadie, UberEats, UberFreight [8,9].

The crowdsourced freight delivery service has evolved to popularize a crowdsourced-driver-only model, which accounts for 60% of Huolala Logistics’ revenue [10]. Under such a system, customers place orders via the client app of an online-to-offline (O2O) freight delivery platform, providing demand information such as call-in time, pickup location, delivery destination, and the volume and weight of the freight. Rather than maintaining a privately owned fleet, the O2O platform dispatches all pickup and delivery tasks to crowdsourced drivers, who share their availability, vehicle type, and current location on the service app. The platform then arranges pickup and delivery times based on its promised service efficiency. Some delivery services promise a pickup within a specific time limit (e.g., 30 minutes), others guarantee delivery within a certain timeframe (e.g., 2 hours), or they offer both. As heterogeneous customer orders continuously arrive, the platform faces the challenge of dynamic dispatching, where it must update its dispatch plan to minimize total service costs while maintaining promised service efficiency. Otherwise, the platform bears the profit losses due to inefficient arrangements, or customers may experience delayed service. Therefore, efficiently dispatching crowdsourced vehicles to customer orders has become an urgent necessity. We define this issue as the crowdsourced freight delivery routing problem (or CFDRP for short).

Note that the CFDRP is both dynamic and stochastic. Its dynamism arises from the presence of pending orders in vehicles when a new assignment arrives. Its randomness, in turn, stems from the stochastic nature of customer demand for pickups and deliveries, which involves variations in order size, call-in time, pickup location and delivery destination.

The CFDRP differs from the well-known meal delivery routing problem [11] in that it must consider customer satisfaction for both pickup and delivery. Due to the high uncertainty and real-time changing demands in the CFDRP, we employ a rolling-horizon approach [12] to manage this complexity. This method allows the platform to continuously update plans in response to the stochastic dynamic environment. Under such an approach, the platform updates its plans based on available information at each decision epoch, rather than attempting to forecast the order arrivals in advance. This approach effectively decomposes the CFDRP into a series of subproblems, each treated as a deterministic pickup and delivery problem at each decision epoch. Note that the master problem remains dynamic and stochastic; the rolling-horizon approach does not transform it into a static-deterministic problem but instead provides a structured way to make decisions under uncertainty. Therefore, we propose a mixed-integer linear programming (MILP) formulation to solve each subproblem. To address each subproblem, we develop two heuristic algorithms to re-optimize the platform’s plan, aiming to minimize the total service costs borne by the platform, which ultimately influences the average charges for customers.

Overall, our main contribution lies in: (1) Introducing a new formulation for the crowdsourced freight delivery routing problem, which integrates pickup and delivery operations into a dynamic vehicle routing problem with crowdsourced drivers, latency penalties, capacity constraints and empty depot considerations, reflecting real-world business models. (2) Incorporating heuristics into rolling-horizon frameworks to solve the problem and developing an improved partheon genetic algorithm. Through numerical experiments in comparisons with simulated annealing, we demonstrate the superiority of our algorithm. (3) Conducting a real-world case study to examine the practical applicability of our approach, the results demonstrate significant improvements in cost reduction and service efficiency.

The remainder of the paper is organized as follows. In Section 2, we briefly review the related literature. In Section 3, we formally describe the crowdsourced freight delivery routing problem, and the novel formulation of this model is provided in Section 4. Due to the complexity of the integer programming formulation, two algorithms are proposed to solve the problem in Section 5. Numerical experiments are presented in Section 6, followed by a real-life case study in Section 7. Finally, we conclude with overall remarks in Section 8.

## 2 Literature review

Our research primarily lies in the literature on crowdsourced freight delivery routing problem, with a particular focus on examining issues related to pickup and delivery pairing and dynamic routing planning. Therefore, we will review the crowdsourced freight delivery routing problem, the pickup and delivery problem, and the dynamic vehicle routing problem sequentially.

### 2.1 Crowdsourced freight delivery routing problem

Motivated by Walmart and Amazon’s “crowdshipping" practices, Archetti et al. [13] examine an early-stage crowdsourced freight delivery service that outsources delivery tasks to in-store customers instead of professional logistics companies. These customers served as occasional drivers, delivering goods along their return routes and earning a commission. Similarly, Macrina et al. [14] introduce a time window and propose two distinct mathematical models for multi-deliveries and split deliveries, respectively. Arslan et al. [15] expand on the framework of Archetti et al. [13] by incorporating multiple pickup locations, thereby increasing the heterogeneity of customer demand. Dayarian and Savelsbergh [16] consider the situation of in-store customers completing same-day home delivery and proposed two solutions based on a rolling-horizon approach: one is a myopic solution, and the other incorporates probabilistic information about in-store customer and online order arrivals. Note that all the papers introduced above assume that drivers are in-store customers willing to make deliveries on their way home. However, this setting is not currently applied in practice, as none of the mainstream e-retailers or courier companies currently operate in this way [8].

As the development of crowdsourced logistics becomes increasingly specialized, we do not consider the case where drivers are in-store customers, focusing on the case where drivers who receive jobs from professional crowdsourced logistics platforms. Qi et al. [17] consider the case where drivers are hired for shared-mobility with flexible working hours, similar to the case in ride-sharing services. This article investigates a network that contains multiple last-mile delivery terminals, that act as transshipment nodes. Inbound deliveries are shipped by the logistics service provider’s trucks, while outbound shipments are completely carried by shared-mobility drivers. Torres et al. [18] investigate the vehicle routing problem with time windows and stochastic supply of crowd-vehicles, considering both professional drivers and crowdsourced drivers. The authors propose a two-stage stochastic model and used a branch-and-price algorithm to solve the problem. Cheng et al. [19] primarily examine how to effectively manage the workforce of crowdsourced delivery platforms to meet changing demands while minimizing the costs of hiring contract workers and crowdsourced couriers under uncertain conditions. The article presents a basic simplified model and a more generalized model, estimating the upper and lower bounds of costs through these two models.

In these studies, crowdsourced drivers serve as either a supplement to a company’s existing logistics capacity or operate in a manner where they can freely reject orders, similar to ride-sharing services. However, as the crowdsourced logistics industry has evolved, platforms relying entirely on crowdsourced capacity have emerged, handling all the pickup and delivery tasks through crowdsourced drivers with their vehicles. Compared with the previous literature, our study adopts two significant assumptions: (1) the platform’s capacity relies solely on crowdsourced vehicles, and (2) drivers are obligated to accept assigned delivery orders, eliminating the flexibility of rejection.

### 2.2 Pickup and delivery problem

The pickup and delivery problem involves a fleet of vehicles that fulfill orders with designated pickup and delivery points. This issue has been extensively documented in the transportation field [20–23]. The pickup and delivery problem has developed rapidly in city logistics and caught the attention of many scholars. For example, Liu et al. [24] address the pickup and delivery problem in home health care logistics by providing two mixed-integer programming models to handle four types of demands, considering time windows. They develop a genetic algorithm and a tabu search method, with computational experiments proving the effectiveness of these methods on an existing benchmark. Crainic et al. [25] study the pickup and delivery problem in city logistics with three types of transportation demands. They develop an enhanced tabu search metaheuristic based on different traffic routes (inbound, outbound, and intra-city traffic). Numerical experiments demonstrate that the metaheuristic’s search strategies significantly enhance the quality of the city logistics system. Steever et al. [26] define the virtual food court delivery problem as a generalization of the meal delivery problem, allowing for multiple pickup locations in one customer order. They propose an auction-based heuristic that utilizes forward-looking metrics, including equity and dispersion, to assess platform preparedness for future demands. Cai et al. [27] focus on the dynamic pickup and delivery problem in the manufacturing industry, specifically optimizing cargo transportation between different factories. The study presents a new model that includes constraints such as docks, time windows, capacity, and last-in-first-out loading. They also introduce a variable neighborhood search algorithm with multiple local search strategies and an efficient disturbance to address this problem. Su et al. [28] examine the dynamic pickup and delivery problem with time windows, heterogeneous vehicles, no depot, and dynamic priority in a shared logistics platform. They propose a mathematical model to maximize profit by considering dynamic customer priorities, where various types of vehicles start from random locations. To effectively solve this complex problem, they introduce a parallel hybrid search algorithm that integrates genetic algorithms, variable neighborhood search, and tabu search, achieving high-quality solutions within a short computational time.

### 2.3 Dynamic vehicle routing problem

The dynamic vehicle routing problem [29,30] involves dispatching vehicles in a system where various parameters, such as the arrival pattern of customers, their location, and demand information, are unknown beforehand but revealed over time. Therefore, vehicle routes should be periodically or continuously re-optimized as new information becomes available. This type of system requires real-time technology assistance between the dispatcher and vehicles for information exchange. Researchers generally adapt the solution developed for the static vehicle routing problem to the dynamic case under the rolling-horizon framework. Ojeda Rios et al. [29] conducted a comprehensive review on this issue. Motivated by their taxonomy, we categorize existing literature in two aspects: *dynamic and deterministic routing problems* and *dynamic and stochastic routing problems*.

For the dynamic and deterministic routing problem, all system parameters are regarded as deterministic, despite some of them having a stochastic nature. Therefore, related studies propose a myopic policy that only considers the current problem state. For example: Santos and Xavier [31] analyze the matching problem in ride-sharing services where money is the incentive. Occasional drivers provide their trip information to the online platform, while passengers share their order information. The objective of the online platform is to match transactions and determine vehicle routes. This paper suggests using a greedy randomized adaptive search procedure within a rolling-horizon framework to solve this issue. Ng et al. [32] investigate an online vehicle routing problem, a formation of the capacitated vehicle routing problem that incorporates rerouting strategies to combat inefficiencies caused by traffic congestion. In response to this issue, vehicles must adjust their routes upon reaching each destination, taking traffic into account to minimize travel time. They use image processing technologies to assess congestion levels and develop the multiple colonies artificial bee colony algorithm for problem-solving purposes.

For the dynamic and stochastic routing problem, some system parameters are treated as stochastic terms. Therefore, related studies propose a proactive policy that not only considers the current state of the problem, but also anticipates the future state of the problem. For example: Ulmer [33] examine how anticipation and reactive reoptimization strategies perform in dynamic vehicle routing problems with stochastic customer requests. This research used mixed-integer programming for reactive re-optimization and approximate dynamic programming for anticipatory optimization, comparing their benefits under different levels of dynamism. Levering et al. [34] discuss how to improve dynamic routing in road networks with changing conditions to minimize vehicle travel time. They introduce a new stochastic process that incorporates intelligent transportation systems data to model traffic uncertainties and a Markov-modulated background process to monitor events that impact travel speeds. They present the Edsger algorithm, which is optimized for real-time use, along with speed-up methods to enhance efficiency.

## 3 Problem description

We define the crowdsourced freight delivery routing problem with online pickup and delivery planning. The problem considers a transportation network comprising a fleet of homogeneous crowdsourced vehicles and a set of heterogeneous customer orders within a specific district. This network includes four types of nodes: initial vehicle nodes, order pickup nodes, order delivery nodes, and final vehicle nodes. Customer orders arrive dynamically over the planning horizon  [ 0 , *H* ] . However, the platform does not have prior knowledge of future customer order arrivals. All order information is revealed in real-time during the planning and execution of vehicle routes.

At each decision epoch within the planning horizon, there is a set of available vehicles *V * in the distribution range, each vehicle has an initial node $\Delta_{v}^{0}$ and a capacity constraint *g*. To ensure completeness of vehicle routes and provide better clarity of the model, we introduce a virtual final vehicle node $\Delta^{*}$ for *v* ∈ *V*, assuming that there is no central depot and that vehicles will receive new assignments in the next decision epoch.

Each customer order *i* has a call-in time $ct_{i}$ and includes information about the cargo $l_{i}$, the pickup node, and the delivery node. Furthermore, an expected service time $s_{i}$ is generated, representing the estimated time required to deliver the order from the pickup node to the delivery node. Upon receiving an order, the platform will provide an estimated pickup time $T_{p}$ and an estimated delivery time $T_{d}$. A vehicle should arrive at any pickup or delivery node before these estimated times; otherwise, penalties will be incurred to the platform.

**Fig 1:**
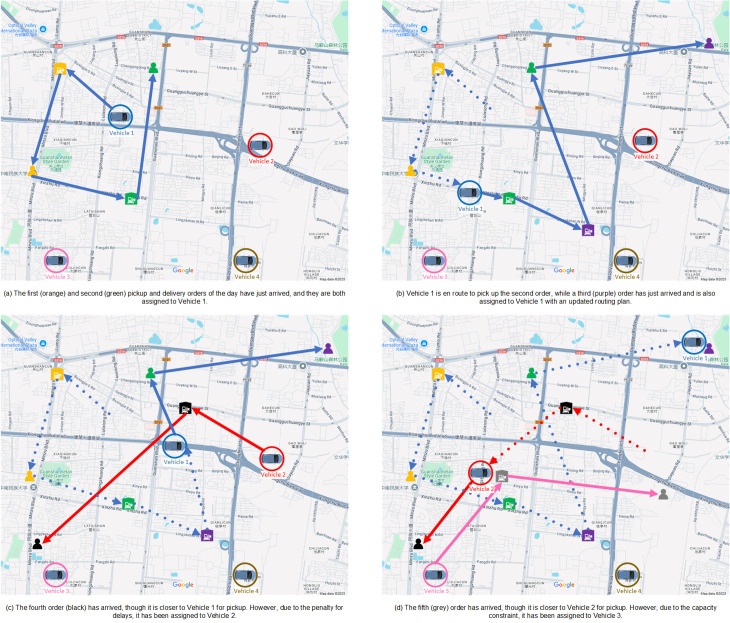
The visualization of the CFDRP. The visualization illustrates key elements of the CFDRP, along with the planning and execution process for a simple example with 5 orders. It features car symbols representing the real-time location of vehicles, squares with forklift icons for pickup points, customer icons for delivery points, solid lines for planned routes, and dashed lines for completed routes.

The problem involves dispatching crowdsourced vehicles to cover all customer orders that arrive randomly over the entire planning horizon, with the objective of minimizing the total service costs. These costs include the dispatched vehicle cost, calculated based on a “basic price and mileage price" pricing model, as well as the penalty cost incurred for lateness in customer service. We have developed a visualization component based on Google Maps to intuitively display the crowdsourced freight delivery routing problem (CFDRP) (see Fig 1).

## 4 Mathematical formulation for the crowdsourced freight delivery routing problem

### 4.1 Online strategy

Many platforms implement a strategy where outsourced drivers must commit their availability in advance and share their real-time vehicle information via an online app. These platforms update the routing plan and mandatory assignment to drivers through the same app, rather than allowing drivers to reject offers as is common in ride-sharing services like Uber and Lyft. We refer to this as the “Online Strategy" for short. We adopt the rolling-horizon approach (RHA), a methodology for managing dynamic systems characterized by high uncertainty [26], to construct a framework for continuously re-optimizing the online strategy.

**Fig 2:**
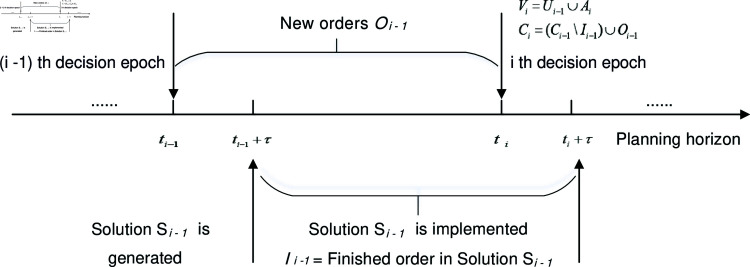
The dynamism of the online strategy. The timeline and the information updating process.

Now, we introduce the sequence of events over the planning horizon  [ 0 , *H* ] , which represents a whole day or a shift according to the business setting. With the rolling-horizon approach, we introduce *M* decision epochs $t_{1},t_{2},\ldots ,t_{M}$ (where $t_{1}=0<\cdots <t_{M}<H$) into the planning horizon. The time interval *h* between two consecutive epochs is constant and prespecified, therefore $t_{i}=(i\;-\;1)\;*\;h$ for  ( *i* = 1 , ⋯ , *M* ) . At each decision epoch $t_{i}$, we solve a static problem based on all the information revealed up to this point in time. After a computing period *τ*, the platform releases the updated solution for pickup & delivery assignment and routing plans to each outsourced vehicle driver, who will execute his respective tasks according to the updated solution during the time interval $\left[t_{i}\;+\;\tau ,t_{i+1}\;+\;\tau \right]$. Fig 2 illustrates the dynamism of this approach.

Next, we detail the static problem faced by the platform upon each decision epoch $t_{i}$ for *i* = 1 , *…* , *M*, where $C_{i}$ and $V_{i}$ represent the set of orders and the set of crowdsourced vehicles at $t_{i}$, respectively. To update the information on orders and vehicles over the planning horizon, we assume that the static problem from the previous decision epoch, $t_{i-1}$, has been solved, with the solution denoted as $S_{i-1}$ (*i* ∈ { 2 , *…* , *M* } ). A set of vehicles, $V_{i-1}$, is dispatched to cover all orders, $C_{i-1}$, and implement the solution $S_{i-1}$ at the implementation time $t_{i-1}\;+\;\tau$. By the next implementation time, $t_{i}\;+\;\tau$, some vehicles may not have completed their assigned pickup or delivery tasks. We denote the set of vehicles with pending orders as $U_{i-1}\subset V_{i-1}$, and the set of pending orders as $K_{i-1}$. At the current decision epoch $t_{i}$, the available vehicles include those that were unassigned in $V_{i-1}$, vehicles that were assigned and have completed all orders by $t_{i}\;+\;\tau$, new vehicles added during the interval $\left[t_{i-1},t_{i}\right]$, and all vehicles in $U_{i-1}$. The first three terms are collectively denoted as $A_{i}$. Similarly, the orders to be matched consist of pending orders in $K_{i-1}$ (with completed orders denoted as $I_{i-1}$) and new orders $O_{i-1}$ that were updated during the time interval $\left[t_{i-1},t_{i}\right]$. Therefore, $V_{i}$ and $C_{i}$ are expressed as:


$$\begin{array}{c} V_{i}=U_{i-1}\cup A_{i}\\ C_{i}=(C_{i-1}\backslash I_{i-1})\cup O_{i-1} \end{array}$$

**Fig 3:**
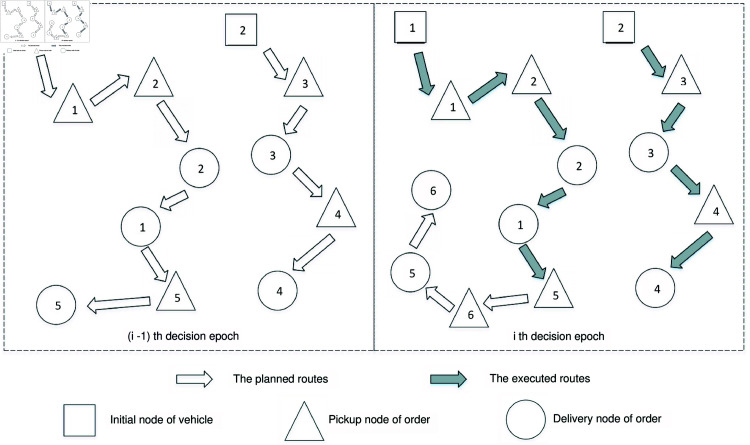
The decision-updating process of the CFDRP.

The static problem for each decision epoch $t_{i}$ involves dispatching a subset of the available crowdsourced vehicles, $V_{i}$, to cover all orders in $C_{i}$, with the objective of minimizing the total service costs over the horizon $\left[t_{i}\;+\;\tau ,H\right]$. We would like to emphasize that the orders in $K_{i-1}$ remain assigned to their respective vehicles, $U_{i-1}$, at each decision epoch $t_{i}$. In other words, the assignment on orders made in the previous decision epochs stays unchanged. However, we can re-optimize the scheduled routes for the pending orders in $K_{i-1}$, along with newly arrived orders. To illustrate the decision-updating process of CFDRP, Fig 3 shows the rules for assignment and re-routing across two consecutive decision epochs.

### 4.2 Mathematical formulation

The CFDRP is essentially a dynamic vehicle routing problem involving pick & delivery operations, an empty depot, outsourced vehicles, and penalty costs for delays. In addition, the following mild assumptions are made based on real-world condition:

- Vehicles must cover all the orders revealed up to the decision epoch in the dynamic system.
- Vehicles are sufficient and uniform in capacity; each must remain available over the entire planning horizon and cannot reject any assignments from the platform.
- Once an assignment is made at a specific decision epoch, it cannot be transferred between vehicles; however, vehicles may receive additional orders and new route instructions at each decision epoch.

To facilitate the formulation of objective functions and constraints, the parameters and decision variable notations are defined as follows. A mixed-integer linear program (MILP) is employed to assign orders and update routes for crowdsourced vehicles, based on the information revealed upon the current decision epoch, including newly received orders and available vehicles.

Parameters:

- *C*: Set of all customers, *C* = { 1 , . . . , *n* } .
- *P*: Set of pickup nodes, *P* = { 1 , . . . , *n* } .
- *D*: Set of delivery nodes, *D* = { *n* + 1 , . . . , 2*n* } .
- *V *: Set of available vehicles, with capacity $\bar{V}$, *V* = { 1 , . . . , *m* } .
- *B*: Set of pickup and delivery nodes, *B* = *P⋃* ⁡ *D*.
- $\Delta_{v}^{0}$: Initial vehicle node, *v* ∈ *V*, $\Delta_{v}^{0}=\{2n+1,2n+2,\cdots \;,2n+v\}$.
- $\Delta^{*}$: A virtual final vehicle node for vehicle v∈V, ensuring zero travel time from the penultimate location of any vehicle to this node.
- *N*: All nodes in the transportation network, $N=\Delta_{v}^{0}⋃B⋃\Delta^{*}$.
- *G*: A graph composed of nodes N and arcs A=N×N, where G=(N,A).
- $N_{v}$: All nodes that vehicle *v* ∈ *V* can serve, $N_{v}=\Delta_{v}^{0}⋃B⋃\Delta^{*}$.
- $G_{v}$: A graph composed of nodes $N_{v}$ and arcs $A_{k}=N_{v}*N_{v}$, where $G_{v}=(N_{v},A_{v})$.
- $C_{v}$: Set of customers assigned to vehicle *v* ∈ *V* in the previous decision epoch without completing the job in that epoch.
- $U_{c}$: Set of vehicles, where each $c\in C_{v}$ has already been assigned.
- $s_{i}$: Service time (loading or unloading) at node i, where i∈N, with $s_{\Delta_{v}^{0}}=s_{\Delta^{*}}=0$.
- *T_i_*: Estimated time it takes to arrive at the node *i* ∈ *B*.
- $q_{i}$: Amount of cargos picked up from node $i\in P(q_{i}>0)$ or dropped off at node $i\in D(q_{i}<0)$.
- $g_{v}$: Amount of cargos being carried by vehicle *v* ∈ *V* at the decision epoch.
- *g*: Capacity constraint of each vehicle.
- $d_{ij}$: Travel distance between node *i* and node *j*, $d_{ij}\geq 0,(i,j)\in A$.
- $t_{ij}$: Travel time between node *i* and node *j*, $t_{ij}\geq 0,(i,j)\in A$.
- *M*: A sufficiently large positive integer.

Decision variables:

- $x_{vij}$: Binary decision variable equals one if vehicle *v* ∈ *V* travels from node *i* to node *j*; otherwise, zero.
- $S_{vi}$: Continuous decision variable representing the time at which vehicle *v* ∈ *V* visits node *i*.
- $L_{vij}$: Continuous decision variable representing the amount of cargos being carried by vehicle *v* ∈ *V* while a route to node *j* after visiting node *i*.

The objective can be characterized as:


$$\begin{array}{c} Min\;\;C_{0}*(\bar{V}-\underset{v\in V}{\sum }x_{v,\Delta_{v}^{0},\Delta^{*}})+C_{1}*\underset{v\in V}{\sum }\underset{(i,j)\in A}{\sum }d_{ij}*x_{vij}+C_{2}*\underset{v\in V}{\sum }\underset{i\in B}{\sum }max(S_{vi}-T_{i},0) \end{array}$$ (1)

and the constraints can be written as:


$$\begin{array}{c} \underset{v\in V}{\sum }\underset{j\in N}{\sum }x_{vij}=1,\forall i\in P. \end{array}$$ (2)

$$\begin{array}{c} \underset{j\in N_{V}}{\sum }x_{vij}-\underset{j\in N_{V}}{\sum }x_{v,j,i+n}=0,\forall v\in V,i\in P. \end{array}$$ (3)

$$\begin{array}{c} \underset{i\in N_{v}}{\sum }x_{vij}=1,\forall j\in C_{v},v\in U_{c}. \end{array}$$ (4)

$$\begin{array}{c} \underset{j\in P⋃\Delta^{*}}{\sum }x_{v,\Delta_{v}^{0},j}=1,\forall v\in V. \end{array}$$ (5)

$$\begin{array}{c} \underset{i\in D⋃\overset{0}{\underset{v}{\Delta }}}{\sum }x_{v,i,\Delta^{*}}=1,\forall v\in V. \end{array}$$ (6)

$$\begin{array}{c} \underset{i\in N_{v}}{\sum }x_{vij}=\underset{k\in N_{v}}{\sum }x_{vjk},\forall v\in V,j\in N_{v}. \end{array}$$ (7)

$$\begin{array}{c} S_{vi}+s_{i}+t_{ij}+M*(x_{vij}-1)\leq S_{vj},\forall v\in V,(i,j)\in A_{v}. \end{array}$$ (8)

$$\begin{array}{c} S_{vi}\leq S_{v,n+i},\forall v\in V,i\in P. \end{array}$$ (9)

$$\begin{array}{c} L_{vij}\leq g,\forall v\in V,(i,j)\in A_{v}. \end{array}$$ (10)

$$\begin{array}{c} L_{v,\Delta_{v}^{0},j}=(g_{v}+q_{j})*x_{v,\Delta_{v}^{0},j},\forall v\in V,j\in N_{v},j\neq \Delta_{v}^{0}. \end{array}$$ (11)

$$\begin{array}{c} L_{v,j}\geq L_{v,i}+q_{i}+M*(x_{vij}-1),\forall v\in V,(i,j)\in A_{v}. \end{array}$$ (12)

$$\begin{array}{c} T_{v,i}\geq 0,\forall v\in V,i\in N_{v}. \end{array}$$ (13)

$$\begin{array}{c} L_{vij}\geq 0,\forall v\in V,(i,j)\in A_{v}. \end{array}$$ (14)

$$\begin{array}{c} x_{vij}\in 0,1,\forall v\in V,i\in \Delta_{vj}^{-},j\in \Delta_{v}^{+}. \end{array}$$ (15)

The objective function (1) aims to minimize the total service costs, which comprise the weighted sum of the vehicles utilized, distance traveled, and penalty costs incurred due to lateness in customer service. Here, $C_{0}$ denotes the unit vehicle cost, $C_{1}$ represents the unit transportation cost, and $C_{2}$ signifies the unit penalty cost.

Constraints (2–4) ensure the fulfillment of pickup and delivery requirements in terms of quantity. Constraint (2) guarantees the visitation of each pickup node, while constraint (3) ensures that the delivery node is visited only if the related pickup node is visited, and both are serviced by the same vehicle. Additionally, constraint (4) dictates that a vehicle must complete any pending pickup or delivery tasks if assigned such orders in prior decision epochs.

Vehicle routing is addressed through constraints (5–7). Constraint (5) mandates that each vehicle departs from its initial node, while constraint (6) requires each vehicle to reach the virtual final node. Furthermore, constraint (7) enforces that each node is visited and left by the same vehicle an equal number of times.

The timing relationships are addressed in constraints (8) and (9). Specifically, constraint (8) states that the sum of the arrival time at node i, the service time at node i, and the travel time from node i to node j must be less than or equal to the arrival time at node j. Constraint (9) ensures that each pickup node is visited before the corresponding delivery node.

Constraints (10–12) govern loading issues. Constraint (10) ensures that the capacity constraints of the vehicles are respected. Constraint (11) defines a vehicle’s load after visiting the first node in current planning route to be their initial load being carried by vehicle at the decision epoch plus the load carried by the vehicle during that first visit. Constraint (12) captures the net load variety. Finally, constraints (13–15) establish the ranges of decision variables.

## 5 Algorithm design

To effectively tackle the subproblem at each decision epoch, we propose an improved partheno genetic algorithm (IPGA) that employs a single chromosome design, unlike conventional dual chromosome setups, thus avoiding the generation of additional infeasible solutions during crossover. Additionally, the IPGA introduces novel crossover operations tailored to paired pickup and delivery orders. The four types of crossover operators (PDDV, PDSV, EUV, IPDV) significantly diversify the population while preserving feasibility. These operators are designed to address the specific challenges of our context, ensuring efficient exploration of the solution space. To evaluate the effectiveness of our IPGA, we also present a simulated annealing (SA) algorithm. The detailed procedures of the two algorithms are as follows.

### 5.1 Improved partheno genetic algorithm

**Fig 4:**
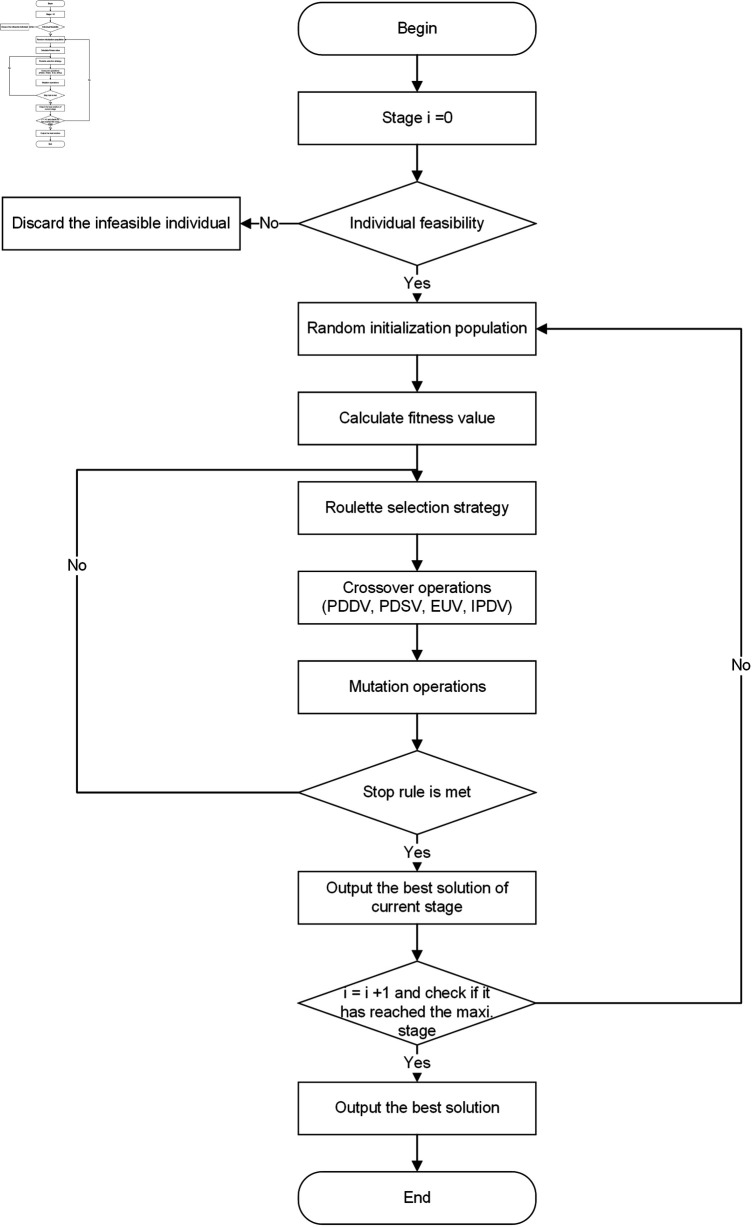
The process of IPGA.

The IPGA includes initial population generation, chromosome representation, fitness function evaluation, crossover operation, and mutation operation. The algorithm’s process is depicted in Fig 4.

**Fig 5:**
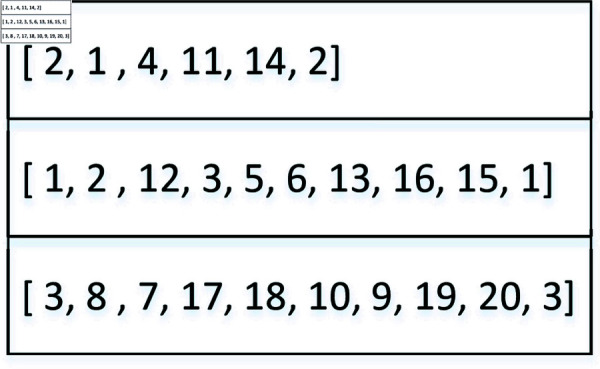
Chromosome representation at first stage.

(1) *Chromosome presentation and population initialization.* Each chromosome represents a solution of vehicle routes broken down into multiple gene segments. Fig 5 illustrates the structure of a chromosome, displaying 10 orders with their corresponding pickup (numbered 1-10) and delivery (numbered 11-20) nodes at the first decision epoch. Each row corresponds to a vehicle route at the current stage, with the first and last numbers in each row indicating the vehicle index. For instance, in the first row, vehicle 2 picks up orders at nodes 1 and 4 then delivers them to nodes 11 and 14 respectively. Nodes 1 and 11, as well as nodes 4 and 14, form a pair for pickup and delivery within the same order.

At the first decision epoch, pickup & delivery points and vehicles are randomly generated. The first vehicle accommodates points until its capacity limit is reached, followed by the allocation of remaining points to subsequent vehicles until all orders are loaded. This forms an initial solution (see Fig 5), and the initial population comprises 500 chromosomes/solutions.

**Fig 6:**
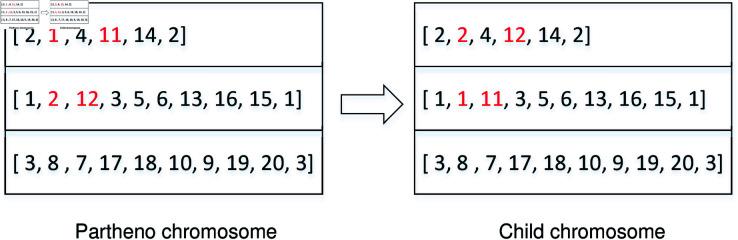
Crossover operation of PDDV.
- *PDSV *. In this operation, a vehicle is selected from a partheno chromosome, and two pairs of pickup and delivery points are randomly chosen. These two pairs of points are then exchanged to form a new vehicle route, as well as a child chromosome (see Fig 7).

(2) *Fitness function evaluation and selection operation.* The total service costs encompasses fixed vehicle costs, travel expenses, and penalties. Each individual’s fitness is calculated using the formula: *fitness value = 1/Objective function*. In addition, a roulette wheel selection strategy is employed for choosing parent chromosomes, where higher fitness values facilitate easier selection.

(3) *Crossover operation.* Crossover operation is a fundamental aspect of genetic algorithms, significantly impacting the algorithm’s effectiveness. Various types of crossover operations exist, which can be classified into four categories: exchanging pickup and delivery pairs between different vehicles (PDDV), swapping pickup or delivery points within the same vehicle (PDSV), interchanging empty and utilized vehicles (EUV), and inserting a pair of pickup and delivery points into another vehicle (IPDV). The specific details of these operations are delineated below.

- *PDDV *. This operation involves selecting two vehicle routes from the partheno chromosome and randomly selecting a pair of pickup and delivery points from each route. Subsequently, the two pairs are exchanged to create a new child chromosome. Fig 6 illustrates the procedures of the PDDV crossover operation, where exchanging order 1 from the first route and order 2 from the second route results in the generation of the new child chromosome.

**Fig 7:**
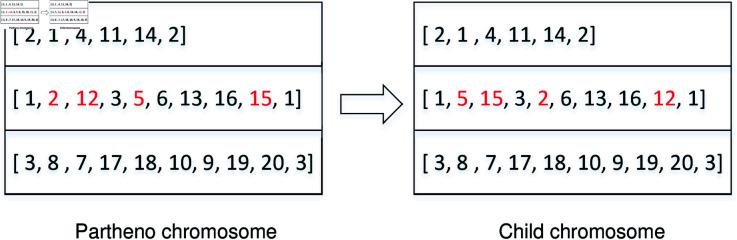
Crossover operation of PDSV.
- *EUV *. Since all vehicles have different initial nodes, by exchanging an empty vehicle with an utilized vehicle, along with the pickup and delivery points, a child chromosome can be created from the partheno chromosome (see Fig 8).

**Fig 8:**
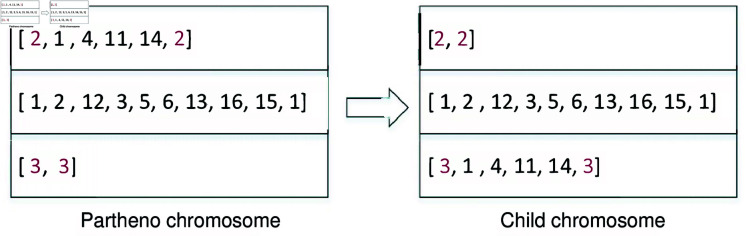
Crossover operation of EUV.
- *IPDV *. This crossover operation consists of two steps: inserting the pickup point and inserting the delivery point.
  Step 1: Select a pair of pickup and delivery points from one utilized vehicle. Next, insert these points into another vehicle using all possible methods (see Step 1 in Fig 9). By maintaining the original sequence of points, the new child chromosome is created. Calculate the fitness values for all insertion methods, retaining only the child chromosome with the largest gap by comparing the fitness values with the partheno chromosome.
  Step 2: Identify the optimal locations of pickup and delivery points in the new child chromosome. Insert the delivery point into all feasible locations behind the pickup point. Re-evaluate the fitness values for all possibilities and retain the chromosome with the largest gap as the final child chromosome (see Step 2 in Fig 9).

**Fig 9:**
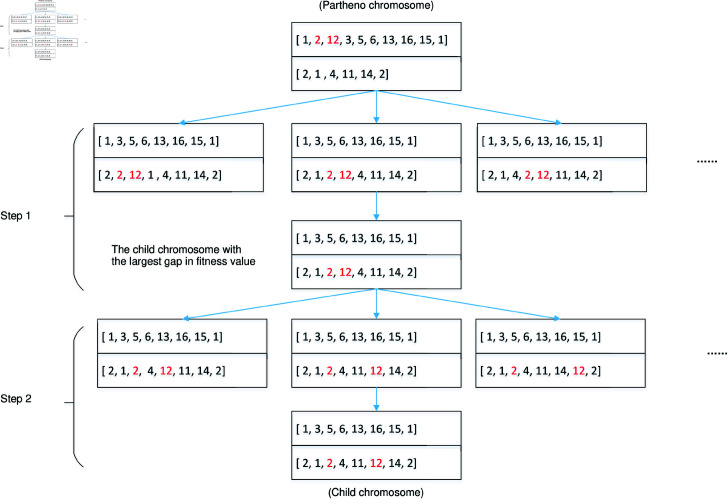
Crossover operation of IPDV.

**Fig 10:**
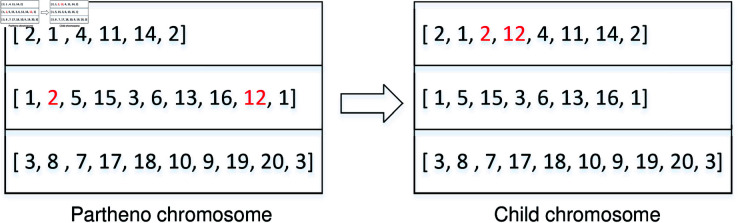
Mutation operation.

(4) *Mutation operation and feasibility verification.* Mutation operations encompass various types, including real value mutation and binary compilation, among others. We adopt real value mutation to enhance the child chromosome. In cases where certain orders incur excessive penalty costs due to extended vehicle routes, inserting the order with the highest penalty cost into an empty vehicle can reduce the total penalties. Fig 10 demonstrates an instance of mutation operation, where inserting order 2 from the second route into the first route effectively mitigates the penalty cost.

After the mutation operations, infeasible solutions may arise when no empty vehicles are available. To address this, the order with the highest penalty cost is inserted into the vehicle with the lowest cost, resulting in a feasible child chromosome.

### 5.2 Simulated annealing

**Fig 11:**
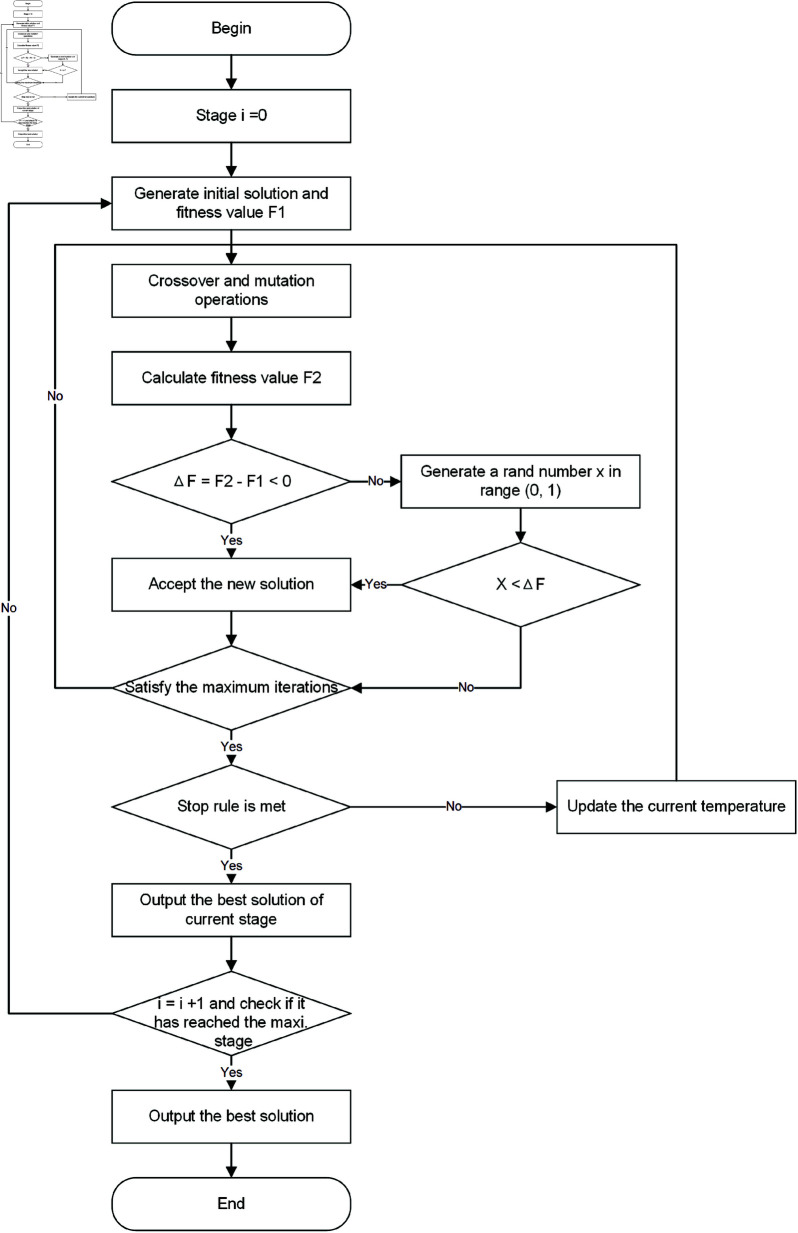
The process of SA.

Simulated annealing (SA) has since been widely utilized for solving optimization problems [35,36]. This study focuses on utilizing SA to analyze pickup and delivery problems. The SA algorithm consists of three main components: the initial solution, crossover and mutation operations, and stop rules (see Fig 11).

*Step 1*: Initialize the parameters including the initial temperature $T_{0}$, stop temperature $T_{1}$, maximum iterations *M* at each temperature, maximum number of iterations *R*, and the annealing rate *Q*, with the current temperature set as $T=T_{0}$.

*Step 2*: Generate the initial solution randomly, similar to the improved genetic algorithm, and calculate the fitness value $F_{1}$. It is important to note that SA retains only one solution, unlike the genetic algorithm which maintains multiple solutions.

*Step 3*: Perform crossover and mutation operations similar to the genetic algorithm to obtain a new solution and its corresponding fitness value $F_{2}$.

*Step 4*: Check if the difference $\Delta F(\Delta F=F_{2}-F_{1})$ is negative; accept the new solution if true, otherwise accept the solution with probability *exp* ( - *ΔF* ∕ *T* ) .

*Step 5*: Repeat steps 2-4 at each temperature, updating *T* = *T* × *Q* after *M* iterations, and continue iterating through steps 2-4.

*Step 6*: Output the best solution if the temperature reaches $T_{1}$ and the number of iterations reaches *R*; otherwise, continue iterating.

## 6 Numerical experiments

As described previously, two algorithms are developed to address this optimization problem. In order to evaluate the effectiveness of the algorithms, we coded the algorithms in MATLAB language on a personal computer (Intel Core i7 CPU, 3.00 GHz; Memory, 8G). According to real-life business operations, we assume that there is no depot and the initial position of vehicles are different and changing over the planning horizon. we set the capacity constraint as 7.2 $m^{3}$, the average traveling speed is 30 *km* ∕ *h*, the unit penalty cost is 10 *rmb* ∕ *h*, the fixed cost is 90 *rmb*, the unit traveling cost is 7.5 *rmb* ∕ *km*. In each decision epoch, the number of orders need to be arranged is the same. For IPGA, we assume the initial population is 500, the crossover rate is 100%, the mutation rate is 0.1 and the maximum iteration is 500. For SA, the initial temperature $T_{0}=500$, stop temperature $T_{1}=0.1$, the maximum iterations M=300 at each temperature, the maximum number of iterations R=500, and the annealing rate *Q* = 0 . 9. Three numerical tests are simulated with different settings of orders and epochs, and the test results are shown in Tables 1, 2, and 3.

**Table 1:** Comparative analysis of total service costs under 3 epochs.

Paired pickup-delivery	Epochs	Total order number	IPGA	$T_{IPGA}$	SA	$T_{SA}$	Gap
5	3	15	2318.19	278	2780.96	332	16.64%
10	3	30	4648.50	291	5439.21	352	14.54%
20	3	60	10475.91	313	11908.61	365	12.03%
30	3	90	16655.59	295	18888.75	318	11.82%
40	3	120	25997.48	320	28989.64	354	10.32%
50	3	150	27949.42	696	31099.12	748	10.13%
Average	/	/	/	/	/	/	12.58%

**Table 2:** Comparative analysis of total service costs under 5 epochs.

Paired pickup-delivery	Epochs	Total order number	IPGA	$T_{IPGA}$	SA	$T_{SA}$	Gap
5	5	25	5023.50	260	5910.35	340	15.01%
10	5	50	10672.05	457	12284.58	546	13.13%
20	5	100	23208.03	674	26029.09	715	10.84%
30	5	150	33701.77	1036	37654.33	1046	10.50%
40	5	200	44694.25	1294	49638.45	1180	9.96%
50	5	250	52931.38	1946	58516.94	1804	9.55%
Average	/	/	/	/	/	/	11.50%

**Table 3:** Comparative analysis of total service costs under 6 epochs.

Paired pickup-delivery	Epochs	Total order number	IPGA	$T_{IPGA}$	SA	$T_{SA}$	Gap
5	6	30	5957.77	320	6944.13	399	14.20%
10	6	60	11910.54	419	13631.29	501	12.62%
20	6	120	26126.07	919	29568.89	1027	11.64%
30	6	180	46758.55	1199	52262.03	1149	10.53%
40	6	240	60505.28	1776	66836.53	1663	9.47%
50	6	300	71393.95	2475	78728.43	2295	9.32%
Average	6	/	/	/	/	/	11.30%

The results, including running times and outcomes for each algorithm, represent the average from 10 trials. For instance, as shown in the third row of Table 2, with an epoch number of 5, we randomly generated 20 orders per epoch, totaling 100 orders to serve. The experiment demonstrates that on average, the IPGA can reduce costs by 10.84% compared to the SA; however, it requires an additional 41 seconds. Therefore, decision-makers should weigh the trade-off between time and cost based on their specific business environment. Furthermore, though the advantage of IPGA over SA diminishes with increasing order numbers, IPGA consistently outperforms SA across various parameter settings, reducing total service costs by more than 10% on average. For instance, total service costs decrease by 12.58% for 3 epochs, 11.50% for 5 epochs, and 11.30% for 6 epochs.

This set of experiments demonstrates that the IPGA significantly outperforms the SA. Relevant platform managers can use our proposed IPGA to optimize CDFRP for order-vehicle matching and vehicle routing.

## 7 Case study

To gain insight into the quality of our solutions to CFDRP instance, we have conducted a case study using the Z enterprise instance set, which is derived from real-world historic data. Z enterprise is a crowdsourced freight delivery service platform which primarily focuses on serving wholesale market merchants in the central part of China, providing line-haul, city logistic (e.g., on-demand delivery) and cold chain logistic. We focus on the less-than-truckload business of city logistic, we select the practical data during the peak period (for 10 days) in a certain district to confirm the superiority and feasibility of our solution and algorithm. These CFDRP instances resemble realistic daily order-arrival patterns and vehicle shifts in areas. The characteristics of orders and vehicles are described as Table 4 (the statistical unit is each decision epoch).

**Table 4:** The characteristic of problem instances.

	The size of orders	The volume of each order ($m^{3}$)	The size of updating vehicles
Average	29.53	1.93	28.40
Standard deviation	3.31	0.72	1.58
Median	28	2	28.50
Maximum	35	3	30
Minimum	25	1	25

For each data set, we make experiment to compare the performance of our proposed solution, an IPGA based on the rolling-horizon framework, with current solution employed by the Z enterprise. We ran a series of cases using realistic parameter settings, including the basic parameters of vehicles and the time interval between two consecutive decision epochs. The basic parameters that directly influence total service costs are given as follows: capacity constraint of each vehicle *g* = 7 . 2, unit vehicle cost $C_{0}=90$, unit transportation cost $C_{1}=7.5$, and unit penalty cost $C_{2}=10$. The interval between two consecutive epochs, denoted by h, determines the time for triggering re-optimization and updating the information. It is set to 1 hour to ensure solution feasibility.

**Fig 12:**
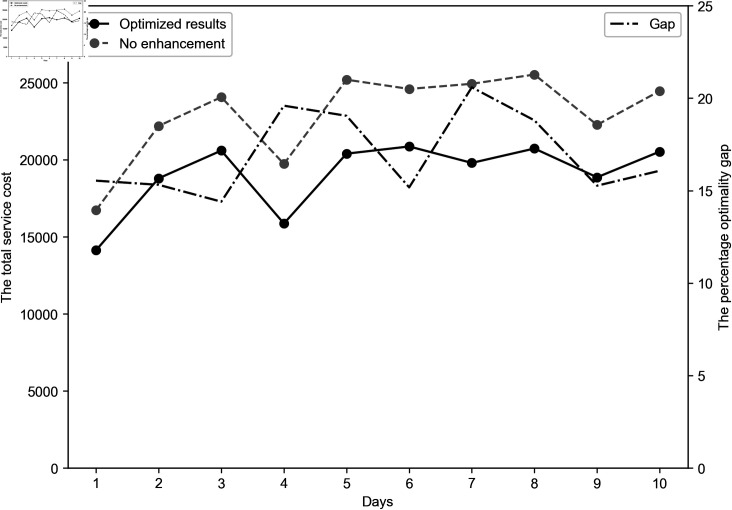
Computational enhancements with IPGA.

The results (see Fig 12) show that the optimized online planning of CFDRP reduces total service costs by an average of 16.90% (up to 20.78%) compared with the “current routing planning without enhancement". This level of cost reduction is significant and can be used to subsidize both drivers and customers, thereby enhancing the platform’s competitiveness in supply and demand markets. In conclusion, our proposed solution demonstrates strong performance in real-world environments.

## 8 Conclusion

This study addresses the online strategy for the crowdsourced freight delivery planning problem and proposes a mixed integer linear programming model based on a rolling-horizon framework, along with an improved partheno genetic algorithm (IPGA) and a simulated annealing algorithm (SA) to solve it. The experimental results show that the IPGA outperforms the SA, reducing the total service costs by over 10% on average. Furthermore, a real-world case study demonstrates the practical applicability of the proposed model and algorithms, providing a solid foundation for real-world implementation. However, we only consider the fixed penalty cost or empty depot. Actually, in addition to outsourced vehicles, there may exist platform-owned vehicles or hard time window. Considering a variety of constraints in the dynamic vehicle routing problem could provide some interesting research directions for future research.
